# Supplementary material for: Measurement of sedentary behaviour in population health surveys: a review and recommendations
Source: PeerJ. 2017 Dec 11;5:e4130. doi: 10.7717/peerj.4130 (PMC5729819; doi:10.7717/peerj.4130)
Supplement: Table S1 [file peerj-05-4130-s001.docx]

**Supplemental table 1.** Sample MEDLINE search strategy

| **Subjective measurement terms** | |
| --- | --- |
| 1 | Self report$.tw. |
| 2 | Questionnaire$.tw. |
| 3 | Diary.tw. |
| 4 | Log$.tw. |
| 5 | Survey.tw. |
| 6 | Interview$.tw. |
| 7 | Recall.tw. |
| 8 | Or/1-7 |
| **Objective measurement terms** | |
| 9 | Acceleromet$.tw. |
| 10 | Activity monitor$.tw. |
| 11 | Activpal.tw. |
| 12 | Calroimet$.tw. |
| 13 | Direct$ observ$.tw. |
| 14 | Or/9-13 |
| **Sedentary terms** | |
| 15 | Sedentary$.tw. |
| 16 | Sitting$.tw. |
| 17 | Tv.tw. |
| 18 | Television.tw. |
| 19 | Car time.tw. |
| 20 | Passive transport$.tw. |
| 21 | Computer.tw. |
| 22 | Screen.tw. |
| 23 | Video gam$.tw. |
| 24 | Or/15-23 |
| **Study design term** | |
| 25 | (reliability or validity).tw. |
| 26 | 8 and 14 and 24 and 25 |
