# Supplementary material for: Measurement of sedentary behaviour in population health surveys: a review and recommendations
Source: PeerJ. 2017 Dec 11;5:e4130. doi: 10.7717/peerj.4130 (PMC5729819; doi:10.7717/peerj.4130)
Supplement: Table S2 — CI, confidence interval; cpm, count per minute; ICC, intraclass correlation coefficient; LoA, limits of agreement; NS, not significant; UK, United Kingdom; USA, United States of America; WHO, World Health Organization. [file peerj-05-4130-s002.docx]

**Supplemental table 2** Sedentary behaviour questions from national population health surveys

| **Survey name, questionnaire name** | **Countries of use, age range** | **Question wording** | **Response options** | **Validity** | **Reliability** |
| --- | --- | --- | --- | --- | --- |
| **Pediatric Population Health Surveys** | | | | | |
| Australian Health Survey, Australian National Nutrition and Physical Activity Survey[63] | Australia  2-4 | 1) What was the total time [child’s name] spent sitting or lying down to watch television, videos or DVDs [yesterday…/on day]? *(asked for each of the past 7 days)*  2) What was the total time [child’s name] spent sitting or lying down to play any video or computer games [yesterday…/on day]? *(asked for each of the past 7 days)*  3) How often is the TV off during meal times?  4) How often is [child’s name] supervised when watching TV?  5) How often is [child’s name] supervised on the Internet or when playing electronic games?  6) How often is the amount of time [child’s name] spends watching TV or using the computer (internet and games) restricted? | 1) Continuous  2) Continuous  3-6) Never, Rarely, Sometimes, Often, Always  Not applicable | Unknown | Unknown |
| Australian Health Survey, Australian National Nutrition and Physical Activity Survey[9] | Australia  5-17 | 1) What was the total time [you/(child’s name)] spent sitting or lying down to watch television, videos or DVDs [yesterday…/on (day)]? *(repeated for each of past 7 days)*  2) What was the total time [you/(child’s name)] spent sitting or lying down to play any Playstation, Nintendo, X-Box, computer or handheld console games [yesterday…/on (day)]? *(repeated for each of past 7 days)*  3) What was the total time [you/(child’s name)] spent sitting or lying down to Use the computer or Internet (excluding games) [yesterday…/on (day)]? *(repeated for each of past 7 days)*  4) How much of this time *[note: this refers back to answer for screen time]* was spent doing homework?  5) How often is the TV off during meal times?  6) How often is homework done before TV/ Playstation©/ Nintendo© are watched or played?  7) How often [are/is] [you/(child’s name)] supervised when watching TV?  8) How often [are/is] [you/(child’s name)] supervised on the Internet or when playing electronic games?  9) How often is the time [you/(child’s name)] [spend/s] watching TV or using the computer restricted? | 1) Continuous  2) Continuous  3) Continuous  4) Continuous  5-9) Never, Rarely, Sometimes, Often, Always  Not applicable | Unknown | Unknown |
| Canadian Assessment of Physical Literacy (CAPL)[64] | Canada, Jersey  8-12 | When answering the following questions, please tell us about what you did LAST WEEK.  1) On a school day, how many hours did you watch TV?  2) On a weekend day, how many hours did you watch TV?  When answering the following questions, please tell us about what you did LAST WEEK.  3) On a school day, how many hours did you play video or computer games or use a computer for something that was not school work?  4) On a weekend day, how many hours did you play video or computer games or use a computer for something that was not school work? | 1 & 2) I did not watch TV on school/weekend days, <1 hour, 1 hour, 2 hours, 3 hours, 4 hours, ≥5 hours  3 & 4) I did not play video/computer games or use a computer other than for school work on school/weekend days, <1 hour, 1 hour, 2 hours, 3 hours, 4 hours, ≥5 hours | Unknown | Unknown |
| Canadian Community Health Survey 2015, Sedentary activities (SAC) [65] | Canada  12-19 | 1) In the last 7 days, how much of your free time did you spend: watching TV, DVDs, movies or Internet videos? *Please do not include time spent watching while exercising.*  2) In the last 7 days, how much of your free time did you spend: playing other video or computer games? *Include games played on a game console, computer or hand-held electronic device such as a tablet or smart phone.*  3) *[Excluding the activities you have already reported, in]* the last 7 days, how much of your free time did you spend on a computer, tablet or smart phone, doing activities such as surfing the Internet, emailing, using Facebook or doing homework?  4) *Now, a few additional questions about activities you do in your leisure time, that is, activities not at work nor during class time nor during transportation.*  *Some of these activities may appear similar, but please only report your activity once.*  In the last 7 days, how much of your free time did you spend: reading books, magazines or newspapers, including in electronic formats? *Include time spent reading as part of your homework, but do not include time spent reading at work, during class time, during transportation or while exercising.* | 1) Min = 0; Max = 95.0  2) Min = 0; Max = 95.0  3) Min = 0; Max = 95.0  4) Min = 0; Max = 95.0 | Unknown | r = 0.04 - 0.13 |
| Canadian Health Measures Survey 2014/2015, Children's Physical Activity (CPA)[66] | Canada  3-11 | 1) On average, about how many hours a day does he watch TV or videos or play video games?  2) On average, about how many hours a day does he watch TV or videos or play video games?  3) On average, about how many hours a day does he spend on a computer, for example, doing homework, playing games, e-mailing, chatting, surfing the Internet? *[Include time spent on the computer with an adult or older child.*  4) On average, about how many hours a day does he watch TV or videos or play video games? *(If under 6, include time spent on the computer with an adult or older child).* | 1, 2, 4) Doesn't watch TV or videos or play video games, <1 hour/day, 1 to <3 hours/day, 4 to <5 hours/day, 5 to <7 hours/day, ≥7 hours/day, don't know, refusal  3) Doesn't use a computer, <1 hour/day, 1 to <3 hours/day, 3 to <5 hours/day, 5 to <7 hours/day, ≥7 hours/day, don't know, refusal | Unknown | Unknown |
| Canadian Health Measures Survey 2014/2015, Sedentary Activities (SAC)[67] | Canada  12-17 | 1) In a typical week in the past 3 months, how much time did you usually spend watching television, DVD's or videos? *Please do not include time spent watching TV or videos on a computer or while exercising*  2) In a typical week in the past 3 months, how much time did you usually spend playing other types of video games on a game console or hand-held electronic device?  3) In a typical week in the past 3 months, how much time did you usually spend on a computer, tablet or iPAD including watching videos, playing computer games, emailing or using the Internet? *Include Internet use on other devices and time spent doing homework on a computer. Do not include time spent on a computer at work or at school.*  4) In a typical week in the past 3 months, how much time did you usually spend reading? *Include time spent reading as part of your homework, but do not include time spent reading at work or at school.* | 1) Min = 0; Max = 95.0  2) Min = 0; Max = 95.0  3) Min = 0; Max = 95.0  4) Min = 0; Max = 95.0 | Unknown | Unknown |
| Children's Leisure Activities Survey (CLASS)[68] | Australia  5-6, 10-12 | During a typical WEEK what other leisure activities do you usually do?  1) TV / videos  2) Playstation / Nintendo / computer games  3) Computer / Internet  4) Reading  5) Travel by car / bus (to and from school)  6) Homework  7) Play indoors with toys  8) Musical instrument  9) Board games/cards  10) Art & craft (e.g. pottery, sewing, drawing)  11) Talk on the phone  12) Listen to music | Total hours/minutes Monday-Friday  Total hours/minutes Saturday & Sunday | Unknown | Unknown |
| COMPASS[69, 70] | Canada, Guatemala  Grades 9-12 | How much time per day do you usually spend in the following activities?  1) Watching/streaming TV shows or movies?  2) Playing video/computer games?  3) Surfing on the internet?  4) Doing homework?  5) Talking on the phone?  6) Texting, messaging, emailing? (Note: 50 texts = 30 minutes) | 0 to 9:45 | Total sedentary (sum of all other sedentary activities)  ICC = 0.15; Cronbach's α = 0.33 | 1) ICC = 0.56; Cronbach's α = 0.74    2) ICC = 0.65; Cronbach's α = 0.79  3) ICC = 0.71; Cronbach's α = 0.84  4) ICC = 0.54; Cronbach's α = 0.72  5) ICC = 0.76; Cronbach's α = 0.86  6) ICC = 0.86; Cronbach's α = 0.93 |
| Health Behaviour in School-Aged Children (2002-2010 )[71] | Belgium, Canada, Switzerland, Czech Republic, Germany, Denmark, Estonia, Spain, Finland, France, Greenland, Greece, Croatia, Hungary, Israel, Italy, Latvia, Macedonia, Netherlands, Norway, Poland, Portugal, Russia, Scotland, Sweden, Slovenia, Ukraine, USA, Wales  11-15 | 1a & b) About how many hours a day do you usually watch television (including DVDs and videos) in your free time? (asked separately for week and weekend days)  2 a & b) About how many hours a day do you usually play games on a computer or games console (PlayStation, Xbox, GameCube etc.) in your free time? (asked separately for weekdays and weekends)  3 a & b) About how many hours a day do you usually use a computer for chatting online, Internet, e-mailing, homework, and so forth in your free time? (asked separately for weekdays and weekends) | 1a & b) Responses were recoded as follows: “none at all” = 0, “about half an hour a day” = .5, “about 1 hour a day” = 1, “about 2 hours a day” = 2, and so forth to “more than 7 hours a day” = 7.5  2a & b) Responses were recoded as follows: “none at all” = 0, “about half an hour a day” = .5, “about 1 hour a day” = 1, “about 2 hours a day” = 2, and so forth to “more than 7 hours a day” = 7.5  3a & b) Responses were recoded as follows: “none at all” = 0, “about half an hour a day” = .5, “about 1 hour a day” = 1, “about 2 hours a day” = 2, and so forth to “more than 7 hours a day” = 7.5 | Validity against 7-day TV viewing diary: Significantly higher report TV viewing vs. diary. | Boys: ICC = 0.76, 95% CI: 0.63, 0.85  Girls: ICC = 0.81, 95% CI: 0.69, 0.88 |
| Health Survey for England, Physical Activity and Sedentary Behavior Assessment Questionnaire (PASBAQ)[43] | England  ≥16 | *Now I’d like to ask you some questions about time that you might have spent sitting down. For these questions, I’d like you to think about what you have done in the last four weeks, that is since (date of interview – 4 weeks) (when you were not doing your (paid) job)...*  1) In the last 4 weeks, how much time did you spend sitting down watching TV (including DVDs and videos) on an average weekday (that is Monday to Friday)?  2) In the last four weeks, how much time did you spend watching TV (including watching DVDs and videos) on an average weekend day (that is Saturday and Sunday)?  3) Looking at show card I, which of these did you do whilst working? Please include any  work you did on weekends.  *CODE ALL THAT APPLY*  4) If 3 = Sit THEN: On an average work day in the last four weeks, how much time did you usually spend sitting down or standing up?  *Now I'd like to ask you some questions about time that you might have spent sitting down. For these questions, I'd like you to think about what you have done in the last four weeks, that is since (date of interview – 4 weeks) (when you were not doing your (paid job)...*  5) In the last four weeks, how much time did you spend sitting down doing any other activity on an average weekday (that is Monday to Friday)? *Please do not include time spent doing these activities while at work.*  6) In the last 4 weeks, how much time did you spend sitting down doing any other activity on an average weekend day (that is Saturday and Sunday)? *Please do not include time spent doing these activities while at work.* | 1) hours and minutes  2) hours and minutes  3) Sitting down or standing up, walking at work (e.g. door to door sales, hospital nurse work), climbing stairs or ladders, lifting, carrying or moving heavy loads  4) Minutes  5) Minutes  6) Minutes | 1/2) **TV** vs. ActiGraph GT1M <50 cpm: women r = 0.16, 95% CI: 0.11-0.22; men r = 0.16, 95% CI:0.10-0.21, vs. <100 cpm: women r = 0.14, 95% CI:0.08, 0.19; men r = 0.13, 95% CI: 0.07-0.19, vs. <200 cpm: women r = 0.11, 95% CI: 0.06-0.17; men r = 0.11, 95% CI: 0.05-0.18  4) **Occupational sitting/standing** vs. <50 cpm: women r = 0.19, 95% CI: 0.11-0.27; men r = 0.17, 95% CI:0.09-0.25,  vs. <100 cpm: women r = 0.20, 95% CI: 0.10-0.30; men r = 0.19, 95% CI: 0.11-0.27, vs. <200 cpm: women r = 0.18, 95% CI: 0.11-0.26; men r = 0.19, 95% CI:0.11-0.27  5/6) **Non-TV sitting** vs. <50 cpm: women r = 0.20, 95% CI: 0.1-0.26; men r = 0.18, 95% CI: 0.12-0.24,  vs. <100 cpm: women r = 0.20, 95% CI: 0.14, 0.25; men r = 0.17, 95% CI: 0.11-0.22, vs. <200 cpm: women r = 0.18, 95% CI: 0.12-0.23; men r = 0.15, 95% CI: 0.09-0.21  **Total sedentary activity** vs. <50 cpm: women r = 0.27, 95% CI:0.22-0.32; men r = 0.26, 95% CI:0.20-0.32, vs. <100 cpm: women r = 0.24, 95% CI:0.19-0.29, men r = 0.23, 95% CI:0.16-0.29, vs. <200 cpm: women r = 0.21, 95% CI:0.16-0.26, men r = 0.20, 95% CI:0.14-0.26 | Unknown |
| International Study of Childhood Obesity, Lifestyle and the Environment (ISCOLE)[72] | Australia, Brazil, Canada, China, UK, Colombia, Finland, India, Kenya, Portugal, South Africa, USA,  9-11 | 1a) On a school day, how many hours did you watch TV?  1b) On a weekend day, how many hours did you watch TV?  2a) On a school day, how many hours per day do you play video or computer games or use a computer for something that was not school work?  2b) On a weekend day, how many hours per day do you play video or computer games or use a computer for something that was not school work? | 1) I did not watch TV, <1 hour/day, 1 hour/day, 2 hours/day, 3 hours/day, 4 hours/day, 5+ hours/day  2) I did not play video games, <1 hour/day, 1 hour/day, 2 hours/day, 3 hours/day, 4 hours/day, 5+ hours/day | Unknown | Unknown |
| Scottish Health Survey (SHeS)[73] | Scotland,  2-15 | 1) Thinking first of weekdays, that is Monday to Friday, how much time on an average day do/does (you/your child) spend sitting watching TV or another type of screen such as a computer, games console or handheld gaming device? *Please do not include any time spent in front of a screen while at school, work or college. INTERVIEWER: Reading a ‘kindle’ or reading on an iPad should not be included here. If the main activity is reading then code under sitting doing other activity such as eating, reading etc.*  2) Now thinking of the weekend, that is Saturday and Sunday, how much time on an average day do/does (you/your child) spend sitting watching TV or another type of screen (such as a computer, games console or handheld gaming device)? *Again, please do not include any time spent in front of a screen while at school, college or work. INTERVIEWER: Reading a ‘kindle’ or reading on an iPad should not be included here. If the main activity is reading then code under sitting doing other activity such as eating, reading etc.*  3) And how much time on an average weekday do/does (you/your child) spend sitting down doing any other activity, such as eating a meal, reading, or listening to music (IF 65+: or napping in a chair)? Please do not include time spent doing these activities while at work.  *INTERVIEWER: examples of these activities include snacking, studying, drawing, doing puzzles/crosswords etc. Do not count time twice e.g. if they watch TV and eat, include that here or at previous question - not both.*  4) And how much time on an average weekend day (that is Saturday and Sunday) do/does (you/your child) spend sitting down doing any other activity, such as eating a meal, reading, or or listening to music or [if over 65] napping in a chair. Please do not include time spent doing these activities while at work. *INTERVIEWER: other examples of these activities include snacking, studying, drawing, doing puzzles/crosswords etc. Do not count time twice e.g. if they watch TV and eat, include that here or at previous question - not both. INTERVIEWER: Time spent reading using a screen (e.g. a kindle or iPad) can be included here is the respondent says reading is the main activity* | 0-13 hours/day *(enter 0 if <1 hour or never watches screen)* | Unknown | Unknown |
| Scottish Health Survey (SHeS)[73] | Scotland, ≥16 | 1) Thinking first of weekdays, that is Monday to Friday, how much time on an average day do you spend sitting watching TV or another type of screen such as a computer, games console or handheld gaming device? Please do not include any time spent in front of a screen while at school, work or college. *INTERVIEWER: Reading a ‘kindle’ or reading on an iPad should not be included here. If the main activity is reading then code under sitting doing other activity such as eating, reading etc.*  2) Now thinking of the weekend, that is Saturday and Sunday, how much time on an average day do you spend sitting watching TV or another type of screen (such as a computer, games console or handheld gaming device)? Again, please do not include any time spent in front of a screen while at school, college or work. *INTERVIEWER: Reading a ‘kindle’ or reading on an iPad should not be included here. If the main activity is reading then code under sitting doing other activity such as eating, reading etc.*  3) When you are at work are you mainly sitting down, standing up or walking about?  4) On an average work day in the last four weeks, how much time did you usually spend sitting down? *INTERVIEWER: if respondent was on holiday or unable to work on any days in the last four weeks, ask them to report the average number of hours on those days they worked.*  5) And how much time on an average weekday do/does (you/your child) spend sitting down doing any other activity, such as eating a meal, reading, or listening to music (IF 65+: or napping in a chair)? Please do not include time spent doing these activities while at work.  *INTERVIEWER: examples of these activities include snacking, studying, drawing, doing puzzles/crosswords etc. Do not count time twice e.g. if they watch tv and eat, include that here or at previous question - not both.*  6) And how much time on an average weekend day (that is Saturday and Sunday) do/does (you/your child) spend sitting down doing any other activity, such as eating a meal, reading, or or listening to music or [if over 65] napping in a chair. Please do not include time spent doing these activities while at work. *INTERVIEWER: other examples of these activities include snacking, studying, drawing, doing puzzles/crosswords etc. Do not count time twice e.g. if they watch TV and eat, include that here or at previous question - not both. INTERVIEWER: Time spent reading using a screen (e.g. a kindle or iPad) can be included here is the respondent says reading is the main activity* | 1) 0-13 hours/day *(enter 0 if less than 1 hour or never watches screen)*  2) 0-13 hours/day *(enter 0 if less than 1 hour or never watches screen)*  3) Sitting down, standing up, walking about, equal time spent doing 2 or more of these  4) Continuous *(enter 0 if less than 1 hour)*  5) 0-13 hours/day *(enter 0 if less than 1 hour or never watches screen)*  6) 0-13 hours/day *(enter 0 if less than 1 hour or never watches screen)* | Unknown | Unknown |
| U.S. National Health and Nutrition Examination Survey [43] | USA, ≥12 | **1999-2000:** Now I will ask about TV watching or computer use. Over the past 30 days, on a typical day how much time altogether did you spend on a typical day sitting and watching TV or videos or using a computer outside of work? Would you say . .  **2000-2001:** Now I will ask about TV watching or computer use. Over the past 30 days, on a typical day how much time altogether did (you/SP) spend on a typical day sitting and watching TV or videos or using a computer outside of work? Would you say . . .  **2007-2008:** The following question is about sitting or reclining at work, at home, or at school. Include time spent sitting at a desk, sitting with friends, traveling in a car, bus, or train, reading, playing cards, watching television, or using a computer. Do not include time spent sleeping. How much time (do you/does SP) usually spend sitting or reclining on a typical day?  **2009-2012:** The following question is about sitting at work, at home, getting to and from places, or with friends, including time spent sitting at a desk, traveling in a car or bus, reading, playing cards, watching television, or using a computer. Do not include time spent sleeping. How much time (do you/does SP) usually spend sitting on a typical day?  **2013-2014:** The following question is about sitting at school, at home, getting to and from places, or with friends including time spent sitting at a desk, traveling in a car or bus, reading, playing cards, watching television, or using a computer. Do not include time spent sleeping. How much time (do you/does SP) usually spend sitting on a typical day? | **1999-2000 & 2000-01)**  <1 hour = 0, 1 hour = 1, 2 hours = 2, 3 hours = 3, 4 hours = 4, ≥5 hours = 5, (you do/s/he does) not watch TV or videos or use a computer outside of work? = 6, refused = 7, don't know = 9  **2007-08, 2009-12, 2013-14:**  Hours and minutes |  |  |
| Youth Risk Behavior Survey[74] | USA, 13-18 | 1) On an average school day, how many hours do you watch TV?  2) On an average school day, how many hours do you play video or computer games or use a computer for something that is not school work? *Count time spent on things such as Xbox, PlayStation, an iPad or other tablet, a Smartphone, texting, YouTube, Instagram, Facebook, or other social media.* | 1) I do not watch TV, <1 hour/day, 1 hour/day, 2 hours/day, 3 hours/day, 4 hours/day, ≥5 hours/day  2) I do not play video or computer games, <1 hour/day, 1 hour/day, 2 hours/day, 3 hours/day, 4 hours/day, ≥5 hours/day | 1) Compared to 7-day log, r = 0.68, p<0.001 | 1) Kappa = 0.47; r = 0.68, p<0.001 |
| Youth Smoking Survey (2008/2009)[75] | Canada, 11-18 | On average, about how many hours a day do you do the following in your free time?  1)Watch TV or videos  2) Play video games  3) Play/surf on a computer  4) Read for fun | None, <1 hour/day, 1 to 2 hours/day, >2 but <5 hours/day, ≥5 hours/day | Unknown | Unknown |
| **Adult Population Health Surveys** | | | | | |
| Australian Diabetes, Obesity, and Lifestyle (AusDiab) Study[46] | Australia, 36-89 | **TV viewing in the last week**  Please estimate the total time during the last week that you spent watching TV or videos/DVDs. This is when it was the main activity that you were doing; for example you would not include time when the television was switched on and you were preparing a meal.  1) Monday to Friday (In hours and/or minutes - fill in all circles on answer sheet).  2) Saturday and Sunday (In hours and/or minutes - fill in all circles on answer sheet)  **Computer, internet, electronic games in the last week**  Please estimate the total time during the last week that you spent sitting and using the computer. For example, include time spent playing any Playstation, Nintendo, X-Box, computer or handheld console games, and time spent on internet activities. Do not include time spent doing paid work on the computer as this should have been included in the previous questions about sitting for work.  3) Monday to Friday (In hours and/or minutes - fill in all circles on answer sheet).  4) Saturday and Sunday (In hours and/or minutes - fill in all circles on answer sheet)  **Sitting for transport in the last week**  Please estimate the total time during the last week that you spent time sitting to travel to or from places? Please include sitting and waiting for transport. Do not include any time you were standing up while travelling or waiting. If you spent sitting for travelling as part of your work, such as being a taxi driver, this should have been included in the question about sitting for work so do not report it here.  5) Monday to Friday (In hours and/or minutes - fill in all circles on answer sheet).  6) Saturday and Sunday (In hours and/or minutes - fill in all circles on answer sheet)  **Sitting for work in the last week**  Please estimate the total time during the last week that you spent sitting down as part of your job while at work or working from home, including meal and snack breaks and sitting to do work such as at desk or in meetings, sitting to use the computer at work, and sitting for travel as part of work such as being a taxi driver?  7) Monday to Friday (In hours and/or minutes - fill in all circles on answer sheet)  8) Saturday and Sunday (In hours and/or minutes - fill in all circles on answer sheet)  **Sitting for other purposes in the last week**  We are interested in any other sitting or lying down that you may have done during your waking hours in the last week that you have not already told us about. For example this could include sitting for reading or hobbies, socialising with friends or family including time on the telephone eating meals; or listening to music. Please estimate the total time during the last week that you spent sitting down NOT including sitting for work, transport, television viewing, or computer use that you have mentioned in the previous answers?  9) Monday to Friday (In hours and/or minutes - fill in all circles on answer sheet).  10) Saturday and Sunday (In hours and/or minutes - fill in all circles on answer sheet) | Hours and minutes | **TV** vs. ActivPAL: overall r = 0.16, 95% CI: 0.09-0.24; weekday r = 0.09, 95% CI: 0.01-0.16; weekend: r = 0.23, 95% CI: 0.16-0.30  **Computer** vs. Activpal: overall r = 0.14, 95% CI: 0.06-0.21; weekday r = 0.12, 95% CI: 0.05-0.19; weekend: r = 0.10, 95% CI: 0.03-0.17  **Transportation** vs. Activpal: overall r = 0.07, 95% CI: -0.01-0.14; weekday r = 0.12, 95% CI: 0.05-0.20; weekend: r = -0.04, 95% CI: -0.11-0.04  **Occupational** vs. Activpal: overall r = 0.25, 95% CI: 0.17-0.31; weekday r = 0.34, 95% CI: 0.27-0.40; weekend: r = 0.07, 95% CI: 0.00-0.14  **Other sitting** vs. Activpal: overall r = 0.06, 95% CI: -0.02-0.13; weekday r = -0.01, 95% CI: -0.09-0.06; weekend: r = 0.11, 95% CI: 0.04-0.18  **Composite of all sitting questions**: r = 0.46, 95% CI: 0.40-0.52 (weaker correlation in oldest group). Mean difference = 2.01 (2.45) hrs/day. Weekday r = 0.49, 95% CI: 0.43-0.54; weekend day r = 0.25, 95% CI: 0.19-0.32 | Unknown |
| Australian Health Survey[76] | Australia, ≥18 | How much time did [you/(selected person name)] spend sitting or lying down to watch television or videos in the last week?  2) How much time did [you/(selected person name)] spend sitting or lying down to play electronic games in the last week?  3) How much time did [you/(selected person name)] spend sitting or lying down to use a computer or the Internet in the last week?  4) How much time did [you/(selected person name)] spend sitting to travel to or from places in the last week?  5) How much time did [you/he/she] spend sitting at work in the last week?  6) How much time did [you/(selected person name)] spend sitting or lying down to other social or leisure activities in the last week?  7) How much time did [you/(selected person name)] spend sitting or lying down to use a phone (e.g. text and talk) in the last week? | 1) Unlimited  2) Unlimited  3) Unlimited  4) Unlimited  5) Hours, minutes, both hours and minutes, did not sit at work in the last week  6) Unlimited  7) Unlimited | Unknown | Unknown |
| Australian National Social Survey[77] | Australia, ≥18 | Do not include time spent at work, during class time or during transportation.  1) Now we'd like you to think about how you travelled from place to place, including to places like work, stores, movies and so on.  During the last 7 days, on how many days did you travel in a motor vehicle like a train, bus, car or tram?  2) How much time did you usually spend on one of those days travelling in a motor vehicle?  Now we would like you to think about the other time that you spent sitting during the last 7 days. Include time at work, at home, while doing course work and during leisure time. This may include time spent sitting at a desk, visiting friends, reading or sitting or lying down to watch television. Do not include any time spent sitting in a motor vehicle that you have already told me about.  3) During the last 7 days, how much time did you usually spend sitting on a WEEK DAY?  4) During the last 7 days, how much time did you usually spend sitting on a WEEKEND DAY? | Continuous; hours and minutes | Unknown | Unknown |
| Canadian Community Health Survey[62] | Canada, ≥12 | In the last 7 days, how much of your free time did you spend:  1) watching TV, DVDs, movies or Internet videos? Please do not include time spent watching while exercising.  2) (In the last 7 days, how much of your free time did you spend): playing other video or computer games? Include games played on a game console, computer or hand-held electronic device such as a tablet or smart phone.  3) [Excluding the activities you have already reported, in] the last 7 days, how much of your free time did you spend on a computer, tablet or smart phone, doing activities such as surfing the Internet, emailing, using Facebook or doing homework?  Now, a few additional questions about activities you do in your leisure time, that is, activities not at work nor during class time nor during transportation. Some of these activities may appear similar, but please only report your activity once.  4) In the last 7 days, how much of your free time did you spend: reading books, magazines or newspapers, including in electronic formats? Include time spent reading as part of your homework, but do not include time spent reading at work, during class time, during transportation or while exercising. | Min = 0; Max = 95.0 | Unknown | Unknown |
| Canadian Health Measures Survey 2014/2015[66] | Canada, 18-79 | 1) In a typical week in the past 3 months, how much time did you usually spend watching television, DVD's or videos? Please do not include time spent watching TV or videos on a computer or while exercising.  2) In a typical week in the past 3 months, how much time did you usually spend playing other types of video games on a game console or hand-held electronic device?  3) In a typical week in the past 3 months, how much time did you usually spend on a computer, tablet or iPAD, including watching videos, playing computer games, emailing or using the Internet? Include Internet use on other devices and time spent doing homework on a computer. Do not include time spent on a computer at work or at school.  4) In a typical week in the past 3 months, how much time did you usually spend on a computer, tablet or iPAD, including watching videos, playing computer games, emailing or using the Internet? Include Internet use on other devices and time spent doing homework on a computer. Do not include time spent on a computer at work or at school.  5) In a typical week in the past 3 months, how much time did you usually spend reading? Include time spent reading as part of your homework, but do not include time spent reading at work or at school. | Min = 0; Max = 95.0 |  |  |
| English Longitudinal Study of Ageing[78] | England, ≥50 | 1) How many hours of television do you watch on an ordinary day or evening, that is, Monday to Friday?  2) How many hours of television do you normally watch in total over the weekend, that is, Saturday and Sunday? | Hours | Unknown | Unknown |
| European Prospective Investigation into Cancer and Nutrition-Potsdam Study (EPIC)[79] | France, Italy, Spain, the UK, Netherlands, Greece, Germany, Denmark, Sweden, Norway, 50-65 | 1) We would like to know the type and amount of physical activity involved in your work. Please check what the best corresponds with your present occupation from the following four possibilities: sedentary occupation, standing occupation, manual work, heavy manual work | 1) sedentary occupation, standing occupation, manual work, heavy manual work | N/A | N/A |
| Health Survey for England, Physical Activity and Sedentary Behaviour Assessment Questionnaire [43] | England, ≥16 | Now I’d like to ask you some questions about time that you might have spent sitting down. For these questions, I’d like you to think about what you have done in the last four weeks, that is since (date of interview – 4 weeks) (when you were not doing your (paid) job)  1) In the last 4 weeks, how much time did you spend sitting down watching TV (including DVDs and videos) on an average weekday (that is Monday to Friday)?  2) In the last four weeks, how much time did you spend watching TV (including watching DVDs and videos) on an average weekend day (that is Saturday and Sunday)?  3) Looking at showcard I, which of these did you do whilst working? Please include any work you did on weekends.  *CODE ALL THAT APPLY*  1 Sitting down or standing up  2 Walking at work (e.g. door to door sales, hospital nurse work)  3 Climbing stairs or ladders  4 Lifting, carrying or moving heavy loads  IF = Sit THEN  On an average work day in the last four weeks, how much time did you usually spend sitting down or standing up?  Now I’d like to ask you some questions about time that you might have spent sitting down. For these questions, I’d like you to think about what you have done in the last four weeks, that is since (date of interview – 4 weeks) (when you were not doing your (paid job)  4) In the last four weeks, how much time did you spend sitting down doing any other activity on an average weekday (that is Monday to Friday)? Please do not include time spent doing these activities while at work.  5) In the last 4 weeks, how much time did you spend sitting down doing any other activity on an average weekend day (that is Saturday and Sunday)? Please do not include time spent doing these activities while at work. | Hours and minutes | **TV viewing** vs. accelerometer with sedentary threshold of <50 cpm: women r = 0.16, 95% CI: 0.11-0.22; men r = 0.16, 95% CI:0.10-0.21, vs. <100 cpm: women r = 0.14, 95% CI:0.08-0.19; men r = 0.13, 95% CI: 0.07-0.19, vs. <200 cpm: women r = 0.11, 95% CI: 0.06-0.17; men r = 0.11, 95% CI: 0.05-0.18  **Occupational** **sitting/standing** vs. <50 cpm: women r = 0.19, 95% CI: 0.11-0.27; men r = 0.17, 95% CI:0.09-0.2; vs. <100 cpm women r = 0.20, 95% CI: 0.10-0.30; men r = 0.19, 95% CI: 0.11-0.27; vs. <200 cpm women r = 0.18, 95% CI: 0.11-0.26; men r = 0.19, 95% CI:0.11-0.27  **Non-television sitting** vs. <50 cpm women r = 0.20, 95% CI: 0.1-0.26; men r = 0.18, 95% CI: 0.12-0.24; vs. <100 cpm: women r = 0.20, 95% CI: 0.14-0.25; men r = 0.17, 95% CI: 0.11-0.22; vs. <200 cpm women r = 0.18, 95% CI: 0.12-0.23; men r = 0.15, 95% CI: 0.09-0.21  **Total sedentary activity** vs. <50 cpm: women r = 0.27, 95% CI:0.22-0.32; men r = 0.26, 95% CI:0.20-0.32, vs. <100 cpm women r = 0.24, 95% CI:0.19-0.29, men r = 0.23, 95% CI:0.16-0.29, vs. <200 cpm women r = 0.21, 95% CI:0.16-0.26, men r = 0.20, 95% CI:0.14-0.26 | Unknown |
| International Physical Activity Questionnaire (IPAQ) - **Short form**[48, 49, 40, 80] | **Study 1:** Australia, Brazil, UK, Canada, Finland, Netherlands, Japan, Portugal, USA, Sweden, South Africa, Guatemala, 18-65[48]  **Study 2:** South Africa, ≥60[49]  **Study 3:** USA, UK, Netherlands (not English), 35.9 (11.3)[40]  **Study 4:** Belgium, France, Finland, Germany, Italy, the Netherlands, Spain, UK, ≥18[80] | These questions are about how you traveled from place to place, including to places like work, stores, movies, and so on.  1) During the last 7 days, on how many days did you travel in a motor vehicle like a train, bus, car, or tram?  2) How much time did you usually spend on one of those days traveling in a train, bus, car, tram, or other kind of motor vehicle?  **Short form:** Now think about the time you spent sitting on week days during the last 7 days. Include time spent at work, at home, while doing course work, and during leisure time. This may include time spent sitting at a desk, visiting friends, reading or sitting or lying down to watch television.  3s) During the last 7 days, how much time did you usually spend sitting on a week day?  4s) During the last 7 days, how much time did you usually spend sitting on a weekend day? | 1) days per week  2) hours and minutes per day  3) Hours and minutes  4) Hours and minutes | **Study 1**: Sitting time compared to CSA accelerometer: Short form past 7-days completed by telephone: Australia r = 0.32; past 7-days completed by self: USA1 r = 0.45; USA2 r = 0.49; UK1 r = 0.25; usual week by telephone: USA2 r = 0.27; usual week self completed: USA1 r = 0.40  **Study 3:** Compared to accelerometer (<100 cpm): r = 0.34; UK r = 0.25; USA1 r = 0.45; USA2 r = 0.49.  **Study 4:** NR | **Study 1**: Short form past 7 days by telephone: UK2 r = 0.50; short form past 7 days completed by self: UK1: r = 0.73; USA1 r = 092; USA2 r = 0.85; USA2 r = 0.71; Short form usual week telephone: USA2 r = 0.73-0.75; Short form usual week self: USA1 r = 0.94  **Study 2**: Men r = 0.76, p = 0.0000; women r = 0.77, p = 0.0000  **Study 3:** UK r=0.73; USA1 r=0.92; USA2 r=0.85  **Study 4:** Weekdays: r = 0.62, weekend: r = 0.46 |
| International Physical Activity Questionnaire (IPAQ) - **Long form[48, 81, 40, 50]** | **Study 1:** Australia, Brazil, UK, Canada, Finland, Netherlands, Japan, Portugal, USA, Sweden, South Africa, Guatemala, 18-65[48]  **Study 2:** Scotland, 18-65[81]  **Study 3:** USA, UK, Netherlands (not English), 35.9 (11.3)[40]  **Study 4**: USA, 44.6 (10.9)[50] | These questions are about how you traveled from place to place, including to places like work, stores, movies, and so on.  1) During the last 7 days, on how many days did you travel in a motor vehicle like a train, bus, car, or tram?  2) How much time did you usually spend on one of those days traveling in a train, bus, car, tram, or other kind of motor vehicle?  **Long form:** The last questions are about the time you spend sitting while at work, at home, while doing course work and during leisure time. This may include time spent sitting at a desk, visiting friends, reading or sitting or lying down to watch television. Do not include any time spent sitting in a motor vehicle that you have already told me about.  3l) During the last 7 days, how much time did you usually spend sitting on a weekday?  4l) During the last 7 days, how much time did you usually spend sitting on a weekend day? | 1) days per week  2) hours and minutes per day  3) Hours and minutes  4) Hours and minutes | **Study 1:** Sitting time compared to CSA accelerometer: Long form past 7-days completed by self (English countries only): UK1 r = 0.25, USA1 r = 0.26; USA2 r = 0.49. Long-form usual week telephone: USA2 r = 0.23. Long-form usual week self completed: USA1 r = 0.22.  **Study 2:** Sitting time compared to thigh-worn activPAL: Total week: r = 0.11, p = 0.35; total week + transportation: r = 0.16, p = 0.19; total weekdays: r = 0.17, p = 0.15; total weekends: r = 0.01, p = 0.94. Large mean difference (2.15-4.64 hours/day) with large LoA and consistent underestimation  **Study 3:** Compared to accelerometer (<100 cpm): Long form: UK: r = 0.24; USA1 r = 0.30; USA2 r = 0.50. Long form + transportation: UK r = 0.25; USA1 r = 0.26; USA2 r = 0.49.  **Study 4:** Compared to ActiGraph GT1M (<100 cpm) Significant difference in mean minutes/day between NHANES accelerometer reduction and IPAQ-L sitting: 667.4 (95% CI: 650, 685) vs. 502.1 (95% CI: 468.7, 535.4) | **Study 1:** Long form past 7 days self: UK1 r = 0.74; USA1 r = 0.89; USA2 r = 0.78-0.85; long form usual week telephone: USA2 r = 0.85-0.87; long form usual week self: USA1 r = 0.93.  **Study 3:** UK r=0.74; USA1 r=0.95; USA2 r=0.85 |
| NIH-AARP Diet and Health Study[82] | USA, ≥50 | 1) During a typical 24-hour period over the past 12-months, how much time did you spend watching television or videos?  2) During a typical 24-hour period over the past 12-months, how much time did you spend sitting? | 1) None, <1 hour, 1-2 hours, 3-4 hours, 5-6 hours, 7-8 hours, ≥9 hours  2) <3 hours, 3-4 hours, 5-6 hours, 7-8 hours, ≥9 hours | Unknown | Unknown |
| Physical Activity and Transit Survey, 2010-2011[83] | USA, ≥18 | 1) On an average day during the last 7 days, from the time you woke up to around 5 o’clock in the evening, how many hours or minutes did you spend sitting?  2) From 5 o’clock in the evening to the time you went to bed on an average day during the last 7 days, how many hours or minutes did you spend sitting? | 1) Hours and minutes  2) Hours and minutes | Vs. ActiGraph GT3x (<100cpm)Total: r = 0.32, p<.001; the correlation was stronger in daytime (r = 0.37, p<.001) versus evening (r = 0.23, p< .001) sitting. Mean difference = 49 mins/day. LoA: −441 to 343 mins/day. Linear regression showed at lower levels of ST, self-report was lower than accelerometer-measured ST. At higher levels of ST, self-report was higher than accelerometer- measured ST (β = 0.59; standard error = 0.02; p< .001; LoA = mean difference ± 200.34). | Unknown |
| Scottish Health Survey[73] | Scotland, ≥16 | 1) Thinking first of weekdays, that is Monday to Friday, how much time on an average day do you spend sitting watching TV or another type of screen such as a computer, games console or handheld gaming device? Please do not include any time spent in front of a screen while at school, work or college. *INTERVIEWER: Reading a ‘kindle’ or reading on an iPad should not be included here. If the main activity is reading then code under sitting doing other activity such as eating, reading etc.*  2) Now thinking of the weekend, that is Saturday and Sunday, how much time on an average day do you spend sitting watching TV or another type of screen (such as a computer, games console or handheld gaming device)? Again, please do not include any time spent in front of a screen while at school, college or work. *INTERVIEWER: Reading a ‘kindle’ or reading on an iPad should not be included here. If the main activity is reading then code under sitting doing other activity such as eating, reading etc.*  3) When you are at work are you mainly sitting down, standing up or walking about?  4) On an average work day in the last four weeks, how much time did you usually spend sitting down? *INTERVIEWER: if respondent was on holiday or unable to work on any days in the last four weeks, ask them to report the average number of hours on those days they worked.*  5) And how much time on an average weekday do/does (you/your child) spend sitting down doing any other activity, such as eating a meal, reading, or listening to music (IF 65+: or napping in a chair)? Please do not include time spent doing these activities while at work.  *INTERVIEWER: examples of these activities include snacking, studying, drawing, doing puzzles/crosswords etc. Do not count time twice e.g. if they watch tv and eat, include that here or at previous question - not both.*  6) And how much time on an average weekend day (that is Saturday and Sunday) do/does (you/your child) spend sitting down doing any other activity, such as eating a meal, reading, or or listening to music or [if over 65] napping in a chair. Please do not include time spent doing these activities while at work. *INTERVIEWER: other examples of these activities include snacking, studying, drawing, doing puzzles/crosswords etc. Do not count time twice e.g. if they watch tv and eat, include that here or at previous question - not both. INTERVIEWER: Time spent reading using a screen (e.g. a kindle or iPad) can be included here is the respondent says reading is the main activity* | 1) 0-13 hours/day *(enter 0 if less than 1 hour or never watches screen)*  2) 0-13 hours/day *(enter 0 if less than 1 hour or never watches screen)*  3) Sitting down, standing up, walking about, equal time spent doing 2 or more of these  4) Continuous *(enter 0 if less than 1 hour)*  5) 0-13 hours/day *(enter 0 if less than 1 hour or never watches screen)*  6) 0-13 hours/day *(enter 0 if less than 1 hour or never watches screen)* |  |  |
| U.S. National Health and Nutrition Examination Survey (NHANES)[84, 85] | USA, ≥20 | 1) Over the past 30 days, on average how many hours per day did you sit and watch TV or videos outside of work?  2) Over the past 30 days, on average about how many hours per day did you use a computer or play computer games outside of work? | 1) None, <1 hour, 1 hour, 2 hours, 3 hours, 4 hours, ≥5 hours  2) None, <1 hour, 1 hour, 2 hours, 3 hours, 4 hours, ≥5 hours | 1) vs. ActiGraph 7164 (<100 cpm) r = 0.22, p<0.001 | Unknown |
| U.S. National Health and Nutrition Examination Survey (NHANES)[84] | USA, ≥12 | 1) 1999-2000: Now I will ask about TV watching or computer use. Over the past 30 days, on a typical day how much time altogether did (you/SP) spend on a typical day sitting and watching TV or videos or using a computer outside of work? Would you say . .  2000-2001: Now I will ask about TV watching or computer use. Over the past 30 days, on a typical day how much time altogether did (you/SP) spend on a typical day sitting and watching TV or videos or using a computer outside of work? Would you say . . .  2) 2007-2008: The following question is about sitting or reclining at work, at home, or at school. Include time spent sitting at a desk, sitting with friends, traveling in a car, bus, or train, reading, playing cards, watching television, or using a computer. Do not include time spent sleeping. How much time (do you/does SP) usually spend sitting or reclining on a typical day?  2009-2012: The following question is about sitting at work, at home, getting to and from places, or with friends, including time spent sitting at a desk, traveling in a car or bus, reading, playing cards, watching television, or using a computer. Do not include time spent sleeping. How much time (do you/does SP) usually spend sitting on a typical day?  2013-2014: The following question is about sitting at school, at home, getting to and from places, or with friends including time spent sitting at a desk, traveling in a car or bus, reading, playing cards, watching television, or using a computer. Do not include time spent sleeping. How much time (do you/does SP) usually spend sitting on a typical day? | 1)  2) Hours and minutes | Unknown | Unknown |
| WHO Global Physical Activity Questionnaire (GPAQ)[86, 21] | Ireland, mean = 44 ± 14 | 1) The following question is about sitting or reclining at work, at home, getting to and from places, or with friends including time spent sitting at a desk, sitting with friends, traveling in car, bus, train, reading, playing cards or watching television, but do not include time spent sleeping. How much time do you usually spend sitting or reclining on a typical day? | 1) Hours and minutes | Overall r = 0.19, p = 0.14. Significant mean difference between GPAQ (300 mins/day) vs. ActiGraph GT3X (696 mins/day) = 348.7 mins/day, p = 0.0001. Wide LoA: -721.1 to +23.7 mins/day. Bias exists with those who were found to be more sedentary less likely to under-report their SB using the GPAQ. | Unknown |
| WHO Global Physical Activity Questionnaire (GPAQ)[86, 22] | USA, 18-65 | 1) The following question is about sitting or reclining at work, at home, getting to and from places, or with friends including time spent sitting at a desk, sitting with friends, traveling in car, bus, train, reading, playing cards or watching television, but do not include time spent sleeping. How much time do you usually spend sitting or reclining on a typical day? | 1) Hours and minutes | Vs. ActiGraph GT1M (<100 cpm) r = -0.12, NS | Short-term (10 days, n = 16) ICC = 0.92, 95% CI: 0.78, 0.97. Long-term (3 months, n = 54); ICC = 0.83, 95% CI: 0.70, 0.90 |
| Welsh Health Survey[87] | Wales  ≥18 | 1) Think about the time you spent sitting on weekdays during the last 7 days. Include time spent at work, at home, while doing course work and during leisure time. This may include time spent sitting at a desk, visiting friends, reading, or sitting or lying down to watch television.  During the last 7 days, how much time did you spend sitting on an average weekday?  2) Think about the time you spent sitting on a weekend during the last 7 days. Include time spent at work, at home, while doing course work and during leisure time. This may include time spent sitting at a desk, visiting friends, reading, or sitting or lying down to watch television.  That weekend, how much time did you spend sitting on an average weekend day? | 1) Hours and minutes  2) Hours and minutes | Unknown | Unknown |

CI = confidence interval, cpm = count per minute, ICC = intraclass correlation coefficient, LoA = limits of agreement, NS = not significant, UK = United Kingdom, USA = United States of America, WHO = World Health Organization
