# Supplementary material for: Measurement of sedentary behaviour in population health surveys: a review and recommendations
Source: PeerJ. 2017 Dec 11;5:e4130. doi: 10.7717/peerj.4130 (PMC5729819; doi:10.7717/peerj.4130)
Supplement: Table S3 — *, Total males and females, separate data not shown; %F, percentage of sample that is female; cpm, counts per minute; ICC, intraclass correlation coefficient; LoA, limits of agreement; N/A, not applicable; NR, not reported; NS, not significant; r, correlation coefficient; SB, sedentary behaviour; SD, standard deviation; UK, United Kingdom; USA, United States of America. [file peerj-05-4130-s003.docx]

**Supplemental Table 3** Sedentary behaviour questionnaires with psychometric testing

| **Questionnaire name** | **Age mean (SD) or range, % F** | **# of SB questions: mode/domain** | **Criterion measure** | **Validity** | **Reliability** |
| --- | --- | --- | --- | --- | --- |
| **Pediatric questionnaires (N = 14)** | | | | | |
| Activity Questionnaire for Adults and Adolescents (AQuAA), (Chinapaw et al., 2009 ) | 12-16 | 4: TV, reading, computer, other | ActiGraph (<699 cpm) | Sum of TV, reading, computer & other: r = 0.23, NS | ICC = 0.57, 95% CI: 0.34, 0.73 |
| Adolescents Sedentary Activities Questionnaire (ASAQ) (Hardy et al. 2007b) | 11-15, 46% | 23: TV, computer, transportation, education, socializing, cultural | N/A | NR | **TV & computer;** total week: ICCs ranged from 0.76, (grade 6 girls) to 0.90 (grade 8 boys and grade 10 girls); weekdays: ICCs ranged from 0.66 (grade 8 girls) to 0.89 (grade 8 boys); weekend days: ICCs ranged from 0.64 (grade 6 girls) to 0.90 (grade 10 girls) (all significant)  **Passive travel;** total week: ICCs ranged from 0.25 (grade 10 boys) to 0.93 (grade 10 girls); weekdays: ICCs ranged from 0.28 (grade 10 boys) to 0.95 (grade 10 girls); weekend days: ICCs ranged from 0.01 (grade 8 boys) to 0.69 (grade 10 girls)  **Educational**: total week: ICCs = 0.54(grade 6 boys) to 0.88 (grade 10 girls); weekdays: ICCs = 0.47 (grade 6 boys) to 0.83 (grade 10 girls); weekend days: ICCs = 0.64 (grade 8 boys) to 0.81 (grade 10 girls).  **Socializing**: Total week: ICCs = 0.42 (grade 8 girls) to 0.81 (grade 6 girls); weekdays: ICCs = 0.20 (grade 8 boys) to 0.74 (grade 6 girls); weekend days: ICCs = 0.34 (grade 10 boys) to 0.74 (grade 8 boys).  **Total of travel, screen, education, cultural, and social:** Total week: ICCs = 0.57 (grade 6 boys) to 0.86 (grade 6 girls); weekdays: ICCs = 0.58 (grade 6 boys) to 0.82 (grade 6 girls); weekend days: ICCs = 0.47 (grade 6 boys) to 0.83 (grade 10 girls). |
| Child Sedentary Activity Questionnaire (CSAQ) (He et al., 2009) | Grades 5-6, 51% | NR: TV, computer, video games | Activity diary | ICC = 0.5 to 0.8 | ICC = 0.98 |
| COMPASS (Leatherdale et al. 2014) | Grades 9-12 | 6: TV, video game, computer, homework, talking on the phone, texting | ActiGraph (<150 cpm) | **Total sum:** ICC = 0.15, r = 0.20, p<0.05 | **TV**: ICC = 0.56; Cronbach's α = 0.74.  **Video games/computer**: ICC = 0.65; Cronbach's α = 0.79.  **Internet**: ICC = 0.71; Cronbach's α = 0.84.  **Homework**: ICC = 0.54; Cronbach's α = 0.72.  **Talking on phone** = ICC 0.76; Cronbach's α = 0.86.  **Texting**: ICC = 0.86; Cronbach's α = 0.93. |
| GEMS Activity Questionnaire, (GAQ) (Treuth et al., 2003) | 9.0 ± 0.6, 100 % | 14: TV, computer/ video games, arts/crafts, board games, homework/ reading, socializing, music | MTI/CSA accelerometer | NR (did not validate against sedentary time from accelerometer) | **TV watching:** yesterday: r = 0.35, usual r = 0.38  **Other sedentary activities:** yesterday r = 0.47, usual r = 0.48 |
| Hardy et al. Sedentary Behavior Questionnaire (Hardy et al., 2007) | 12-15, 100% | 13 | MTI accelerometer | Mean weekly difference (h/wk) between self-report and accelerometer-based measures of SB = 3.2 h/wk ± 11.9 h/wk | NR |
| Health, Eating and Play Study (HEAPS) (Salmon et al., 2006) | 5-6 (parent proxy), 10-12, 50% | 2: TV | NR | NR | ICC = 0.78, 95% CI: 0.69, 0.84 |
| KidActive-Q (Bonn et al. 2012) | 4.2 (1.3), 50% | 1: TV | NR | NR | **Watching TV:** ICC = 0.85, 95% CI: 0.72, 0.97 |
| Netherlands Physical Activity Questionnaire (NPAQ) (Janz et al. 2005) | 4-7, 55% | 1: TV | MTI (for physical activity) | NR | r = 0.68, Kappa = 0.53, 95% CI: 0.33, 0.74 |
| Pre-School Aged Physical Activity Questionnaire (Pre-PAQ) (Dwyer et al. 2011) | 3.0-5.9, 48% | 3: TV, computer, video games, transportation | NR | NR | **TV/video**: ICC = 0.70-0.88  **Computer/electronic games**: ICC = 0.82-0.85  **Transportation**: ICC = 0.31-0.63 |
| Self-Assessed Physical Activity Checklist (SAPAC) (Brown & Holland 2004, Affuso et al. 2011) | **Study 1:** 11.7 (0.5), 50%  **Study 2:** 11- 15, 64% | 4: TV/video, video/ computer games | **Study 2:** ActiGraph  7164 (<100 cpm) | **Study 2:** overall SB [adjusted for total minutes of activity]: r = 0.18, 95% CI: 0.07, 0.28 [r = 0.23, 95% CI: 0.12, 0.33] | **Study 1:** Boys: TV/video ICC = 0.20, computer ICC = 0.40, total ICC = 0.36. Girls: TV/video ICC = 0.38, computer ICC = 0.35, total ICC = 0.34 |
| Taras et al. questionnaire (Taras et al. 1989) | 3-8 (parent proxy), % NR | 3: TV | NR | NR | r = 0.80, p<0.001 |
| Youth Activity Profile (YAP) from the Youth Physical Activity Measurement Study (Saint-Maurice & Welk 2015) | Grades 4-12 | 5: TV, video games, computer, phone, iPad, homework, music, resting | Sensewear Armband | Composite score sum of TV, computer and cell phone time: r = 0.75, p< .001 | NR |
| Youth Risk Behavior Survey (Schmitz et al. 2004, Brener et al. 2002) | **Study 1:**  13-18, 53%  **Study 2:** 11-15 | 1: TV viewing question  TV, computer | **Study 2:** TV & computer diary | **Study 1:** NR  **Study 2**  **TV:** r = 0.46, mean difference = -0.04 h | **Study 1:**  **TV**: Kappa = 0.47  **Study 2:**  T**V:** Kappa = 0.55, r = 0.68 |
| **Adult questionnaires (N = 34)** | | | | | |
| Active-Q (Bonn et al. 2015) | 33-86, 0% | 7: transport, leisure, occupational, TV, reading, computer | GENEA Accelerometer | r = 0.19, 95% CI: 0.04, 0.34 | ICC = 0.80, 95% CI: 0.74, 0.86 |
| Activity Questionnaire for Adults and Adolescents (AQuAA) (Chinapaw et al. 2009, Oostdam et al. 2013) | **Study 1:** 28.9 (3.5), 48%  **Study 2:** 31.4 (3.9), 100% | 4: TV, reading, computer, other sitting | **Study 1:** ActiGraph 7164 (<699 cpm)  **Study 2:** Actitrainer (<700 cpm) | **Summation of TV, reading, computer and other**  **Study 1**: r = 0.15, NS  **Study 2:** r = 0.12 to 0.23, NS, depending on time-point. | **Study 1:** ICC = 0.60, 95% CI: 0.40, 0.74  **Study 2**: NR |
| Australian Diabetes, Obesity and Lifestyle Study (AusDiab) sitting questions (Clark et al. 2015) | 36-89, 55% | 10: transport, occupation, TV, computer/ internet/ electronics, other | activPAL | **Total sum:** mean difference = 2.01 (SD=2.45) h/day, r = 0.46, 95% CI: 0.40, 0.52  **Occupation**: ρ = 0.25, 95% CI: 0.17, 0.31  **Transport**: ρ = 0.07, 95% CI: -0.01, 0.14  **TV**: ρ = 0.16, 95% CI: 0.09, 0.24  **Computer**: ρ = 0.14, 95% CI: 0.06, 0.21  **Other**: ρ = 0.06, 95% CI: -0.02, 0.13 | NR |
| Australian Women's Activity Survey (AWAS) (Fjeldsoe et al. 2009) | 32 (5), 100% | 15: transport, occupation, all-day sitting, domestic | ActiGraph (<100 cpm) | **Sitting**: r = 0.32, p = 0.006 | **Sitting**: ICC = 0.42, 95% CI: 0.13, 0.64 |
| Clemes et al, sitting questionnaire (Clemes et al. 2012) | 41.5 (12.8), 70% | 10: transport, occupation, TV, computer at home, leisure time (separate weekday and weekend day) | ActiGraph (<100 cpm) | **Weekday total:** mean difference = -13.7 mins/day, 95% CI: -69.2, -41.8, r = 0.54, p<.001, ICC = 0.64, p<.001  **Weekend day total:** mean difference = -4.2 mins/day, 95% CI: -91.7, -83.4, r = 0.13, p = 0.41, ICC = 0.20, p = 0.23 | NR |
| Community Health Activities Model Program for Seniors (CHAMPS) (Gennuso et al. 2015, Hekler et al. 2012) | **Study 1:** ≥65, 79%  **Study 2:** 75.3 (6.8), 56% | 9: socializing, church, computer, arts & crafts, entertainment, musical instrument, reading | ActiGraph (<100 cpm) | **Study 1:** Lin's accordance correlation coefficient = 0.005, 95% CI: -0.01, 0.02 (poor validity)  r = 0.14, p = 0.28  Mean difference = 5.21 h/day, 95% CI: 2.2, 8.3  **Study 2**: r = 0.12, p<0.001 | **Study 1:** ICC = 0.64, p<0.001  **Study 2:** ICC = 0.56, 95% CI: NR |
| Domain-Specific Last 7-d Sedentary Time Questionnaire (SIT-Q-7d) (Wijndaele et al. 2014) | 50.3 (7.4), English 54% | 20: TV, computer, screen, reading, transport, occupation, meals, hobbies, social, music, household, care providing | NR | NR | **English sample only:**  **TV**: ICC = 0.69, 95% CI: 0.62, 0.75  **Computer**: ICC = 0.57, 95% CI: 0.48, 0.64  **Total screen:** ICC = 0.61, 95% CI: 0.53, 0.67  **Reading**: ICC = 0.59, 95% CI: 0.51, 0.66  **Transport**: ICC = 0.50, 95% CI: 0.40, 0.58  **Occupation**: ICC = 0.74, 95% CI: 0.67, 0.80  **Meals**: ICC = 0.76; 95% CI: 0.71, 0.81  **Hobbies**: ICC = 0.28, 95% CI: 0.16, 0.38  **Socializing**: ICC = 0.39, 95% CI: 0.29, 0.49  **Listen to music**: ICC = 0.48, 95% CI: 0.38, 0.57  **Household**: ICC = 0.06, 95% CI: -0.06, 0.17  **Care providing**: ICC = 0.63, 95% CI: 0.55, 0.70  **Total**: ICC = 0.53, 95% CI: 0.44, 0.62 |
| Gennuso Sedentary Behaviour Questionnaire (Gennuso et al. 2016) | 70 (8), 64% | 7: TV, computer, reading, socializing, transportation, hobbies, other activities | activPAL | **Total**: r = 0.06, p=0.72  Mean difference = 0.31 hour/day, 95% CI: -6.74, 7.37 | **Total**: ICC = 0.48, p<0.001  **Transport**: ICC = 0.14, p=0.16  **Socializing**: ICC = 0.29, p=0.02  **TV**: ICC = 0.74, p<0001  **Computer**: ICC = 0.93, p<0.001 |
| Global Physical Activity Questionnaire (GPAQ) (Cleland et al. 2014, Herrmann et al. 2013) | **Study 1**: 44 (14), 46%  **Study 2:** 18-65, 50-83% | 1: occupation, socializing, TV, reading, transportation, playing cards | **Study 1:** ActiGraph GT3X (≤100 cpm)  **Study 2:** ActiGraph GT1M (<100 cpm) | **Study 1:** Overall r = 0.19, p = 0.135. Mean difference = 348.7 mins/day, p = 0.0001. LoA = -721.1 to +23.7 mins/day. Bias exists with those who were found to be more sedentary less likely to under-report their SB using the GPAQ.  **Study 2**: r = -0.12, NS | **Study 1:** NR  **Study 2:** Short-term (10 days, n = 16) ICC = 0.92, 95% CI: 0.78, 0.97. Long-term (3 months, n = 54); ICC = 0.83, 95% CI: 0.70, 0.90 |
| International Physical Activity Questionnaire (IPAQ) (Rosenberg et al. 2008, Craig et al. 2003, Kolbe-Alexander et al. 2006, Umstattd et al. 2013) | **Study 1:** 18-65, 35%-75%  **Study 2:** 68 (5), 57%  **Study 3:** 35.9 (11.3), 55%  **Study 4:** 44.6 (10.9), 78% | 4: transport, all-day weekday and weekend sitting | **Study 1:** CSA accelerometer (<100 cpm)  **Study 2:** NA  **Study 3:** CSA 7164 (<100 cpm)  **Study 4:** ActiGraph GT1M (<100 cpm) | **Study 1:** Sitting time completed by telephone: Australia r = 0.32; past 7-days completed by self: USA1 r = 0.45; USA2 r = 0.49; UK1 r = 0.25; usual week by telephone: USA2 r = 0.27; usual week self completed: USA1 r = 0.40  **Study 2:** NR  **Study 3*:**  **Long form sitting:** UK r = 0.24; Netherlands r = 0.26; USA1 r = 0.30; USA2 r = 0.50; total r = 0.33  **Long form sitting + transportation:** UK r = 0.25; Netherlands r = 0.35; USA1 r = 0.26; USA2 r = 0.49; total r = 0.31  **Short form sitting:** UK r = 0.25; Netherlands r = 0.22; USA1 r = 0.45; USA2 r = 0.49; total r = 0.34  **Study 4:**  Significant difference between ActiGraph (667.4 mins/day) vs. IPAQ (502.1 mins/day), p<0.001 | **Study 1**: Sitting past 7 days by telephone: UK2 r = 0.50; completed by self: UK1: r = 0.73; USA1 r = 092; USA2 r = 0.85; USA2 r = 0.71; usual week telephone: USA2 r = 0.73-0.75; usual week self: USA1 r = 0.94  **Study 2**: Men r = 0.76, p = 0.0000; women r = 0.77, p = 0.0000  **Study 3*:**  **Weekday sitting:** UK r = 0.72; Netherlands r = 0.96; USA1 r = 0.95; USA2 r = 0.82; total r = 0.81.  **Weekend sitting:** UK r = 0.64; Netherlands r = 0.96; USA1 r = 0.97; USA 2 r = 0.78; total r = 0.84  **Total sitting:** UK r = 0.75; Netherlands r = 0.96; USA1 r = 0.96; USA2 r = 0.87; total r = 0.82  **Transport:** UK r = 0.81; Netherlands r = 0.93; USA1 r = 0.84; USA2 r = 0.91; total r = 0.84  **Sitting + transport:** UK r = 0.74; Netherlands r = 0.87; USA1 r = 0.95; USA2 r = 0.85; total r = 0.81  **Study 4:** NR |
| Jefferis Sedentary Behaviour Questionnaire (Jefferis et al. 2016) | 71-93, 0% | 4: TV, reading, computer, transportation | ActiGraph GT3X (<100 cpm) | **Total**: r = 0.18, p<0.001, mean difference = 300 mins/day, 95% CI: 291, 309, LoA = -6 to 607  **TV:** r = 0.17, p>0.001, mean difference = 440 mins/day, 95% CI: 433, 447; LoA = 193 – 687 | NR |
| Longitudinal Aging Study Amsterdam (LASA) Questionnaire (Visser & Koster 2013) | 65-92, 49.4% | 20: TV, read, music, hobbies, occupation, socializing, resting, computer, transportation, church or movie theatre, administrative tasks | ActiGraph GT3X (<100 cpm) | **Napping:** r = 0.11, NS  **Reading:** r = 0.21, NS  **Listening to music:** r = 0.14, NS  **TV:** r = 0.22, NS  **Computer:** r = 0.04, NS  **Working:** r = 0.002, NS  **Hobby:** r = 0.20, NS  **Socializing:** r = 0.05, NS  **Transportation:** r = -0.06, NS  **Church/theatre:** r = -0.19, NS  **Total:** r = 0.35, p<0.05 | **Total**: ICC **=** 0.71, 95% CI: 0.57, 0.81 |
| Madras Diabetes Research Foundation - Physical Activity Questionnaire (MPAQ) (Anjana et al. 2015) | 32 (8.7) 48% | 16: TV, prayer, movies, yoga as relaxation, chatting, reading,  sitting, listening to  music etc., passive | ActiGraph GT3X (<100 cpm) | **Total:** r **=** 0.48, 95% CI: 0.32, 0.62  mean bias = 44.4 mins/week, ±2SD −1599 to 1688] mins/week | **Sitting:** ICC = 0.81, 95% CI: 0.78, 0.84  **TV:** ICC = 0.67, 95% CI: 0.61, 0.71 |
| Marshall Sitting Time Questionnaire (Marshall et al. 2010) | 45-63, 62% | 10: TV, computer, transportation, occupation, leisure | ActiGraph GT1M (<100 cpm) and log book | **Total sitting:** women: weekday: mean difference = -63.6, 95% CI: -115.1, -12.07; weekend-day sitting (mean difference = 10.8, 95%  CI: -52.6, 74.2. Not valid in men against accelerometer. | **TV:** Women: weekday: mean diff between T1 and T2 = -18.5, 95% CI: -28.1, -9.0, r = 0.79; weekend day: mean diff = -4.2, 95% CI: -20.9, 12.6, r = 0.57. Men: weekday: mean diff = -11.2, 95% CI: -26.0, 3.7, ICC = 0.65, r = 0.82, weekend day: mean diff = 3.4, 95% CI: -15.8, 22.5, ICC = 0.62, r = 0.66  **Computer**: Women: weekday: mean diff between T1 and T2 = -0.7, 95% CI: -0.91, 7.7, ICC = 0.63, r = 0.80; weekend day: mean diff = 6.3, 95% CI: -1.6, 14.2, ICC = 0.72, r = 0.74. Men: weekday: mean diff = -6.5, 95% CI: -19.0, 5.9, ICC = 0.62, r = 0.78, weekend day: mean diff = 11.7, 95% CI: -3.5, 26.8, ICC = 0.59, r = 0.68.  **Transportation**: Women: Weekday: mean diff between T1 and T2 = -16.7, 95% CI: -26.6, -6.8, r = 0.43; Weekend day: mean diff = -15.3, 95% CI: -26.6, -0.38, r = 0.31. Men: Weekday: mean diff = -13.0, 95% CI: -24.7, -1.2, r = 0.60, Weekend day: mean diff = -2.0, 95% CI: -16.4, 12.5, r = 0.40.  **Occupation**: Women: weekday: mean diff between T1 and T2 = -3.9, 95% CI: -22.6, 14.7, ICC = 0.79, r = 0.81; weekend day: mean diff = -5.6, 95% CI: -15.2, 14.0, r = 0.53. Men: weekday: mean diff = 4.3, 95% CI: -23.4, 14.9, ICC = 0.86, r = 0.84, weekend day: mean diff = -8.1, 95% CI: -27.4, 11.2, r = 0.23  **Leisure time:** Women: weekday: mean diff between T1 and T2 = -1.0, 95% CI: -17.3, 15.4, r = 0.34; weekend day: mean diff = 0.9, 95% CI: -19.2, 21.0, r = 0.31. Men: weekday: mean diff = 6.5, 95% CI: -5.6, 18.5, r = 0.38, weekend day: mean diff = -12.6, 95% CI: -37.0, 11.8, r = 0.32 |
| Measure of Older Adults' Sedentary Time (MOST). Adapted from Salmon Questionnaire (Gardiner et al. 2011) | 73 (8), 73% | 7: TV, computer, reading, hobbies, social | ActiGraph GT1M (<100cpm) | **Total sum:** r = 0.30, 95% CI: 0.02, 0.54. Mean difference = 3.60 h/day with wide LoA +/- 3.82 h/day | **TV**: r = 0.78, 95% CI: 0.63, 0.89; ICC = 0.76, 95% CI: 0.62, 0.86  **Computer**: r = 0.90, 95% CI: 0.83, 0.94; ICC = 0.79, 95% CI: 0.65, 0.88  **Reading**: r = 0.77, 95% CI: 0.62, 0.86; ICC = 0.74, 95% CI: 0.51, 0.86.  **Transport**: r = 0.45, 95% CI: 0.19, 0.65; ICC = 0.40, 95% CI: 0.14, 0.61  **Hobbies**: r = 0.61, 95% CI: 0.39, 0.76; ICC = 0.35, 95% CI: 0.07, 0.58.  **Socializing**: r = 0.38, 95% CI: 0.11, 0.60; ICC = 0.38, 95% CI: 0.11, 0.60 |
| Modified MONICA Optional Study on Physical Activity Questionnaire (MOSPA-Q) (Chau et al. 2012) | >18, 61% | 1: sitting | ActiGraph (<100 cpm) | r = 0.52, p<0.01 | ICC = 0.54, 95% CI: 0.36, 0.68 |
| Multimedia Activity Recall for Children and Adults (MARCA) (Gomersall et al. 2011, Gomersall et al. 2015) | **Study 1:** 31.7 (12.1), 63%  **Study 2:** 28 (7.4), 48% | Unclear (daily recall of all activities): screen time | **Study 1:** NA  **Study 2:** activPAL | **Study 1:** NR  **Study 2:** r = 0.77, 95% CI: 0.64, 0.86; *P* < .001. Bland-Altman analyses revealed a mean bias of +0.59 h/day, LoA: –2.35 hr to +3.53 h/day. | **Study 1:** ICC = 0.99, 95% CI: 0.98, 0.995  **Study 2:** NR |
| Occupational Sitting and Physical Activity Questionnaire (OSPAQ) (Chau et al. 2012, van Nassau et al. 2015, Wick et al. 2016) | **Study 1:** >18, 61%  **Study 2:** 38(11), 86%  **Study 3:** 40.8 (11.4), 79% | 3: occupational sitting | **Study 1:** ActiGraph (<100 cpm)  **Study 2:** activPAL  **Study 3:** ActiGraph (<100 cpm) | **Study 1**: r = 0.65, p<0.01  Mean difference = 22 mins, 95% CI: 3, 41 mins  **Study 2**: r = 0.35 to 0.48, p<0.05  **Study 3:** ICC = 0.51, 95% CI: 0.24, 0.71, mean difference = -3.9 % of day. | **Study 1:** ICC = 0.89, 95% CI: 0.83, 0.92  **Study 2:** NR  **Study 3:** NR |
| Past-day Adults' Sedentary Time (PAST) (Clark et al. 2013) | 33-75, 100% | 7: TV, computer, reading, transport, occupation, hobbies | activPAL | **Total sum:** r = 0.57, 95% CI: 0.39, 0.71. Mean difference = -0.15 hour, LoA: -4.90, 4.60, 95% CI: -0.72, 0.42 | **TV**: r = 0.38, 95% CI: 0.18, 0.55  **Computer**: r = 0.40, 95% CI: 0.21, 0.57  **Reading**: r = 0.37, 95% CI: 0.17, 0.54  **Transport**: r = 0.44, 95% CI: 0.25, 0.60  **Occupation**: r = 0.64, 95% CI: 0.49, 0.75  **Hobbies**: r = 0.36, 95% CI: 0.16, 0.53 |
| Past-day Adults' Sedentary Time-University (PAST-U) (Clark et al. 2016) | 18-55, 47% | 9: TV, computer, reading, transport, occupation, social | activPAL | **Total sum**: r = 0.63, 95% CI: 0.44, 0.76, mean difference = 0.08h, LoA = -3.92 to 4.07h. Lower mean difference among students: -0.02h | ICC = 0.64, 95% CI: 0.45, 0.77 |
| Past-Week Modifiable Activity Questionnaire (PWMAQ) (Pettee et al. 2011) | 52.6 (5.4), 100% | 1: TV | NR | NR | ICC = 0.77, 95% CI: 0.57, 0.82 |
| Past Year Physical Activity Questionnaire (Orsini et al. 2008) | 56-75, 100% | 1: leisure reading/TV | 7-day PA record | Concordance correlation = 0.47, 95% CI: 0.36, 0.69 | NR |
| Physical Activity and Transit (PAT) Survey (Yi et al. 2015) | ≥18, 59% | 2: sitting | ActiGraph GT3X (<100 cpm) | **Total**: r = 0.32, p< .001; **daytime**: r = 0.37, p< .001, **evening**: r = 0.23, p< .001. Mean difference = 49 mins/day. LoA = -441 to 343 mins/day. Linear regression showed at lower levels of ST, self-report < accelerometer-measured ST. At higher levels of ST, self-report > accelerometer-measured ST (β = 0.59; standard error = 0.02; p< .001; LoA = mean difference ± 200.34). | NR |
| Previous Day Recall (Matthews et al. 2013) | 41.3 (14.8), 54% F | Unclear: all domain | activPAL | Men: r = 0.67, p<0.05; mean difference = 0.72 h/day; LoA = -2.61 to 4.05  Women: r = 0.34, p<0.05; mean differences = 0.75 hrs/day; LoA = -2.21 to 3.74 | NR |
| Rapid Assessment Disuse Index (RADI) (Shuval et al. 2014) | 40-79, % NR | 3: sitting | ActiGraph GT3X (<100 cpm) | **Past week:** r = 0.291, p<0.01  **Past month:** r = 0.189, p<0.05  **Past year:** r = 0.245, p<0.01 | **Week:** ICC = 0.56, 95% CI: 0.44, 0.66  **Month:** ICC = 0.58, 95% CI: 0.46, 0.67  **Year:** ICC = 0.60, 95% CI: 0.49, 0.69 |
| Salmon Sedentary Behaviour Questionnaire (Salmon et al. 2003, Gardiner et al. 2011) | **Study 1:** reliability: 23% , 50.8 (13.5); validity: 51% , 38.8 (15.0)  **Study 2:** 73 (8), 73% | **Study 1:** unknown (~9)  **Study 2**: 7: TV, computer, reading, transportation, hobbies, socializing, telephone, listening to music | **Study 1:** 3-day log  **Study 2:** ActiGraph GT1M (<100 cpm) | **Study 1:**  **Computer**: r = 0.6, p<0.01  **Going for a drive, listening to music, hobbies, talking on the telephone, and reading**: r = 0.4, p<.01  **TV and sitting socializing:** r = 0.3, p<.01  **Reading:** r = 0.2, p<0.05  **Study 2:**  **Total**: r = 0.30, 95% CI: 0.02, 0.54; mean difference = 3.60 h/day, LoA = mean difference ± 3.82 h). | **Study 1:**  **TV**: ICC = 0.82, 95% CI: 0.75, 0.87  **Sitting socializing**: ICC = 0.76, 95% CI: 0.66, 0.82  **Reading**: ICC = 0.78, 95% CI: 0.69, 0.84  **Relaxing/resting**: ICC = 0.56, 95% CI: 0.39, 0.68  **Listening to music**: ICC = 0.37, 95% CI: 0.23, 0.50  **Hobby**: ICC = 0.23, 95% CI: 0.07, 0.44  **Going for a drive**: ICC = 0.85, 95% CI: 0.79, 0.89  **Computer**: ICC = 0.62, 95% CI: 0.48, 0.73  **Talking on telephone**: ICC = 0.06, 95% CI: 0.13, 0.19  **Total SB**: ICC = 0.79, 95% CI: 0.71, 0.85  **Study 2:**  **TV**: ICC = 0.76, 95% CI: 0.62, 0.86  **Computer**: ICC = 0.79, 95% CI: 0.65, 0.88  **Reading**: ICC = 0.74, 95% CI: 0.51, 0.86  **Socializing**: ICC = 0.38, 95% CI: 0.11, 0.60  **Transport**: ICC = 0.40, 95% CI: 0.14, 0.61  **Hobbies**: ICC = 0.35, 95% CI: 0.07, 0.58  **Other**: ICC = 0.04, 95% CI: 0.25, 0.32  **Total SB**: ICC = 0.52, 95% CI: 0.27, 0.70 |
| Sedentary Behavior Questionnaire (SBQ) (Rosenberg et al. 2010) | ≥18, 0 to 100% (3 samples) | 18: TV, video games, reading, transport, paperwork, listening to music, telephone, arts and crafts, musical instruments | ActiGraph 7164 (<100 cpm) | **TV**: women r = 0.12, p = 0.04; men r = -0.001,p = 0.99  **Video games**: women r = 0.04, p = 0.49, men r = 0.01, p = 0.84  **Reading**: women r = 0.04, p = 0.49, men r = 0.01, p = 0.84  **Transport**: women r = -0.04, p = 0.47; men r = 0.03, p = 0.60  **Paperwork**: women r = 0.17, p = 0.002, men r = 0.003, p = 0.95  **Total sum**: women r = 0.10, p = 0.07, men r = -0.01, p = 0.81. **Weekday**: women r = 0.06, p = 0.32, men: r = -0.02, p = 0.78. **Weekend day**: women r = 0.18, p = 0.002, men r = -0.005, p = 0.93 | **TV**: weekday: ICC = 0.86, 95% CI: 0.76, 0.92, r = 0.87; weekend: ICC = 0.83, 95% CI: 0.72, 0.90, r = 0.85  **Video games**: weekday: ICC = 0.83, 95% CI: 0.72, 0.90, r = 0.80; weekend: ICC = 0.830, 95% CI: 0.67, 0.88, r = 0.81  **Reading**: weekday: ICC = 0.64, 95% CI: 0.44, 0.78, r = 0.48; weekend: ICC = 0.64, 95% CI: 0.24, 0.67, r = 0.59  **Transport**: weekday: ICC = 0.76, 95% CI: 0.61, 0.86, r = 0.72; weekend: ICC = 0.76, 95% CI: 0.56, 0.83, r = 0.75  **Paperwork**: weekday: ICC = 0.77, 95% CI: 0.63, 0.87, r = 0.64; weekend: ICC = 0.67, 95% CI: 0.44, 0.61, r = 0.64  **Arts & crafts:** weekday: ICC = 0.70, 95% CI: 0.53, 0.82; weekend: ICC = 0.51, 95% CI: 0.27, 0.69  **Sitting musical instrument:** weekday: ICC = 0.90, 95% CI: 0.82, 0.94; weekend: ICC = 0.93, 95% CI: 0.87, 0.96  **Talking on phone:** weekday: ICC = 0.81, 95% CI: 0.68, 0.89; weekend: ICC = 0.73, 95% CI: 0.57, 0.84  **Listening to music:** weekday: ICC = 0.71, 95% CI: 0.54, 0.82; weekend: ICC = 0.67, 95% CI: 0.49, 0.80  **Total sum**: weekday: ICC = 0.85, 95% CI: 0.75, 0.91, r = 0.77; weekend: ICC = 0.79, 95% CI: 0.63, 0.86, r = 0.74 |
| Sedentary, Transportation and Activity Questionnaire (STAQ) (Mensah et al. 2016) | 20-65, 53% | 7: TV, computer, leisure, transport, sitting | ActiGraph GT3X (<150 cpm) | **Total sum**: r = 0.54, p<0.0001 | **TV**: ICC = 0.79, 95% CI: 0.61, 0.89  **Computer**: ICC = 0.64, 95% CI: 0.38, 0.80  **Leisure screen**: ICC = 0.26, 95% CI: -0.08, 0.55  **Transport**: ICC = 0.28, 95% CI: -0.06, 0.56  **Occupation**: ICC = 0.71, 95% CI: 0.49, 0.84  **Leisure sitting time**: ICC = 0.37, 95% CI: 0.03, 0.62;  **Leisure total**: ICC = 0.64, 95% CI: 0.38, 0.80  **Total screen time:** ICC = 0.70, 95% CI: 0.48, 0.84 |
| Aguilar-Farias Single question to assess sitting time (Aguilar-Farias et al. 2015) | 74.5 (7.6), 52% | 1: sitting (weekday, weekend day, previous day) | activPAL | r = 0.13-0.33 | ICC = 0.64-0.79, 95% CI: NR |
| SIT-Q (Lynch et al. 2014) | 34 males: 51.2 (6.7), 47 females: 45.9 (8.6) | 28: past-year sleeping and napping, meals, transportation, work/study/volunteering, childcare/eldercare, light leisure/relaxing | NR | NR | **Meals:** ICC = 0.60, 95% CI: 0.42, 0.74  weekday only ICC = 0.65, 95% CI: 0.48, 0.77  weekend only ICC = 0.41, 95% CI: 0.18, 0.59  **Transportation:** ICC = 0.59, 95% CI: 0.41, 0.73  weekday only ICC = 0.65, 95% CI: 0.48, 0.77  weekend only ICC = 0.51, 95% CI: 0.30, 0.67  **Work, study & volunteering:** ICC = 0.86, 95% CI: 0.78, 0.91  **Childcare/elder care:** ICC = 0.59, 95% CI: 0.40, 0.73  weekday only ICC = 0.60, 95% CI: 0.41, 0.73  weekend only ICC = 0.59, 95% CI: 0.40, 0.73  **Television viewing time:** ICC = 0.84, 95% CI: 0.75, 0.90  weekday only ICC = 0.82, 95% CI: 0.72, 0.89  weekend only ICC = 0.69, 95% CI: 0.53, 0.80  **Computer use at home:** ICC = 0.31, 95% CI: 0.07, 0.52  weekday only ICC = 0.25, 95% CI: 0, 0.47  weekend only ICC = 0.42, 95% CI: 0.19, 0.60  **Leisure time:** ICC = 0.61, 95% CI: 0.43, 0.74  weekday only ICC = 0.63, 95% CI: 0.45, 0.76  weekend only ICC = 0.51, 95% CI: 0.31, 0.68  **Total daily sitting:** ICC = 0.65, 95% CI: 0.49, 0.78 |
| Workforce Sitting Questionnaire (Chau et al. 2011) | 40-59, 63% | 11: occupation, transport, TV, computer, leisure | AcitGraph GT1M (<100 cpm) | **Occupational**: r = 0.45, p<0.01  **Total all domains workday**: r = 0.34, p<0.01  **Total all domains non-workday**: r = 0.23, p<0.05  **Average total work and non-workdays**: r = 0.40, p<0.01 | **Workday**  **Transport**: ICC = 0.67, 95% CI: 0.54, 0.77  **Occupational**: ICC = 0.63, 95% CI: 0.49, 0.74  **TV**: ICC = 0.91, 95% CI: 0.87, 0.94  **Computer at home**: ICC = 0.56, 95% CI: 0.40, 0.69  **Other leisure activities**: ICC = 0.68, 95% CI: 0.55, 0.78  **Total**: ICC = 0.65, 95% CI: 0.51, 0.75  **Non-workday**  **Transport**: ICC = 0.60, 95% CI: 0.45, 0.72  **Occupational**: ICC = 0.50, 95% CI: 0.33, 0.64  **TV**: ICC = 0.79, 95% CI: 0.69, 0.85  **Computer at home**: ICC = 0.81, 95% CI: 0.73, 0.87  **Other leisure activities**: ICC = 0.59, 95% CI: 0.44, 0.71  **Total**: ICC = 0.80, 95% CI: 0.72, 0.87  *Lower reliability among men.* |
| Workplace Computer Use Questionnaire (Douwes et al. 2007) | 25-55, 53% | 2: occupational computer use | Direct observation | r = 0.41; p = 0.001 | NR |
| Workplace sitting questionnaire (Stand Up Australia Study) (van Nassau et al. 2015, Clark et al. 2011) | **Study 1:** 18-65, 60%  **Study 2:** 38(11), 86% | 2: occupational sitting and breaks from sitting | **Study 1:** Accelerometer (brand not specified, <100 cpm)  **Study 2:** activPAL | **Study 1:**  **Sitting time:** r(pearson) = 0.39, 95% CI: 0.22, 0.53; r(spearman) = 0.29, 95% CI: 0.11, 0.44  Mean difference = -2.75 + (0.47 x average), LoA = mean difference ± 2.25 h  **Breaks in sitting time:** r = 0.26, 95% CI: 0.11, 0.44  **Study 2:** r = 0.25 to 0.30, NS | NR |
| Yale Physical Activity Survey for Older Adults (YPAS) (Gennuso et al. 2015) | 75.1 (6.5), 79% | 1: sitting | ActiGraph (<100 cpm) | 8.6% agreement | ICC = 0.59, p<0.001 |

* - Total males and females, separate data not shown, %F - percentage of sample that is female, cpm - counts per minute, h – hour(s), ICC - intraclass correlation coefficient, LoA - limits of agreement, N/A - not applicable, NR - not reported, NS - not significant, r - correlation coefficient, SB - sedentary behaviour, SD - standard deviation, UK - United Kingdom, USA - United States of America

**References:**

Affuso O, Stevens J, Catellier D, McMurray RG, Ward DS, Lytle L, Sothern MS, Young DR. 2011. Validity of self-reported leisure-time sedentary behavior in adolescents. Journal of Negative Results in Biomedicine 10:2 DOI 10.1186/1477-5751-10-2.

Aguilar-Farias N, Brown WJ, Olds TS, Geeske Peeters GM. 2015. Validity of self-report methods for measuring sedentary behaviour in older adults. Journal of Science and Medicine in Sports 18(6):662-666 DOI 10.1016/j.jsams.2014.08.004.

Anjana RM, Sudha V, Lakshmipriya N, Subhashini S, Pradeepa R, Geetha L, Bai MR, Gayathri R, Deepa M, Unnikrishnan R, Binu VSN, Kurpad AV, Mohan V. 2015. Reliability and validity of a new physical activity questionnaire for India. International Journal of Behavioural Nutrition and Physical Activity 12:40 DOI 10.1186/s12966-015-0196-2.

Bonn SE, Bergman P, Trolle Lagerros Y, Sjolander A, Balter K. 2015. A validation study of the web-based physical activity questionnaire active-Q against the GENEA accelerometer. JMIR Research Protocols 4(3):e86 DOI 10.2196/resprot.3896.

Bonn SE, Surkan PJ, Lagerros YT, Balter K. 2012. Feasibility of a novel web-based physical activity questionnaire for young children. Pediatric Reports 4(4):e37 DOI 10.4081/pr.2012.e37.

Brener ND, Kann L, McManus T, Kinchen SA, Sundberg EC, Ross JG. 2002. Reliability of the 1999 youth risk behavior survey questionnaire. Journal of Adolescent Health 31(4):336-342 DOI 10.1016/S1054-139X(02)00339-7.

Brown TD, Holland BV. 2004. Test-retest reliability of the self-assessed physical activity checklist. Perceptual and Motor Skills 99(3 Pt 2):1099-1102 DOI 10.2466/pms.99.3f.1099-1102.

Chau JY, Van der Ploeg HP, Dunn S, Kurko J, Bauman AE. 2011. A tool for measuring workers' sitting time by domain: the Workforce Sitting Questionnaire. British Journal of Sports Medicine 45(15):1216-1222 DOI 10.1136/bjsports-2011-090214.

Chau JY, Van Der Ploeg HP, Dunn S, Kurko J, Bauman AE. 2012. Validity of the occupational sitting and physical activity questionnaire. Medicine and Science in Sports and Exercise 44(1):118-25 DOI 10.1249/MSS.0b013e3182251060.

Chinapaw MJ, Slootmaker SM, Schuit AJ, van Zuidam M, van Mechelen W. Reliability and validity of the Activity Questionnaire for Adults and Adolescents (AQuAA). BMC Medical Research Methodology. 2009;9:58. DOI 10.1186/1471-2288-9-58.

Clark BK, Thorp AA, Winkler EA, Gardiner PA, Healy GN, Owen N, Dunstan DW. 2011. Validity of self-reported measures of workplace sitting time and breaks in sitting time. Medicine and Science in Sports and Exercise 43(10):1907-1912

DOI 10.1249/MSS.0b013e31821820a2.

Clark BK, Winkler E, Healy GN, Gardiner PG, Dunstan DW, Owen N, Reeves MM. 2013. Adults' past-day recall of sedentary time:

reliability, validity, and responsiveness. Medicine and Science in Sports and Exercise 45(6):1198-207 DOI

10.1249/MSS.0b013e3182837f5

Clark BK, Lynch BM, Winkler EA, Gardiner PA, Healy GN, Dunstan DW, Owen N. 2015. Validity of a multi-context sitting questionnaire across demographically diverse population groups: AusDiab3. International Journal of Behavioral Nutrition and Physical Activity 12:148.

Clark BK, Pavey TG, Lim RF, Gomersall SR, Brown WJ. 2016. Past-day recall of sedentary time: Validity of a self-reported measure of sedentary time in a university population. Journal of Science and Medicine in Sport 19(3):237-41 DOI 10.1016/j.jsams.2015.02.001.

Cleland CL, Hunter RF, Kee F, Cupples ME, Sallis JF, Tully MA. 2014. Validity of the global physical activity questionnaire (GPAQ) in assessing levels and change in moderate-vigorous physical activity and sedentary behaviour. BMC Public Health 14:1255.

Clemes SA, David BM, Yi Z, Xu H, Brown W. 2012. Validity of two self-report measures of sitting time. Journal of Physical Activity and Health 9(4):533-539 DOI 10.1123/jpah.9.4.533.

Craig CL, Marshall AL, Sjostrom M, Bauman AE, Booth ML, Ainsworth BE et al. 2003. International physical activity questionnaire: 12-country reliability and validity. Medicine and Science in Sports and Exercise 35(8):1381-95. DOI 10.1249/01.mss.0000078924.61453.fb.

Douwes M, de Kraker H, Blatter BM. 2007. Validity of two methods to assess computer use: Self-report by questionnaire and computer use software. International Journal of Industrial Ergonomics 37(5):425-31 DOI <http://dx.doi.org/10.1016/j.ergon.2007.01.002>.

Dwyer GM, Hardy LL, Peat JK, Baur LA. 2011. The validity and reliability of a home environment preschool-age physical activity questionnaire (Pre-PAQ). International Journal of Behavioral Nutrition and Physical Activity 8:86. DOI 10.1186/1479-5868-8-86.

Fjeldsoe BS, Marshall AL, Miller YD. 2009. Measurement properties of the Australian Women's Activity Survey. Medicine and Science in Sports and Exercise 41(5):1020-1033 DOI 10.1249/MSS.0b013e31819461c2.

Gardiner PA, Clark BK, Healy GN, Eakin EG, Winkler EA, Owen N. 2011. Measuring older adults' sedentary time: reliability, validity, and responsiveness. Medicine and Science in Sports and Exercise 43(11):2127-33 DOI 10.1249/MSS.0b013e31821b94f7.

Gennuso KP, Matthews CE, Colbert LH. 2015. Reliability and validity of 2 self-report measures to assess sedentary behavior in older adults. Journal of Physical Activity and Health 12(5):727-32. DOI 10.1123/jpah.2013-0546.

Gennuso KP, Thraen-Borowski KM, Gangnon RE, Colbert LH. 2016. Patterns of sedentary behavior and physical function in older

adults. Aging Clin Exp Res 28(5):943-50 DOI 10.1007/s40520-015-0386-4.

Gomersall SR, Olds TS, Ridley K. 2011. Development and evaluation of an adult use-of time instrument with an energy expenditure focus. Journal of Science and Medicine in Sport 14(2):143-148 DOI 10.1016/j.jsams.2010.08.006.

Gomersall SR, Pavey TG, Clark BK, Jasman A, Brown WJ. 2015. Validity of a self-report recall tool for estimating sedentary behavior in adults. Journal of Physical Activity & Health 12(11):1485-1491 DOI 10.1123/jpah.2014-0602.

Hardy LL, Bass SL, Booth ML. 2007. Changes in sedentary behavior among adolescent girls: a 2.5-year prospective cohort study. Journal of Adolescent Health 40(2):158-165 DOI 10.1016/j.jadohealth.2006.09.009.

Hardy LL, Booth ML, Okely AD. 2007. The reliability of the Adolescent Sedentary Activity Questionnaire (ASAQ). Preventive Medicine 45(1):71-4. DOI:10.1016/j.ypmed.2007.03.014.

He M, Harris S, Piche L, Beynon C. 2009. Understanding screen-related sedentary behavior and its contributing factors among school-aged children: a socialecologic exploration. American Journal of Health Promotion 23(5):299-308 DOI 10.4278/ajhp.07070965.

Hekler EB, Buman MP, Haskell WL, Conway TL, Cain KL, Sallis JF, Saelens BE, Frank LD, Kerr J, King AC. 2012. Reliability and validity of CHAMPS self-reported sedentary-to-vigorous intensity physical activity in older adults. Journal of Physical Activity & Health 9(2):225-236 DOI 10.1123/jpah.9.2.225.

Herrmann SD, Heumann KJ, Der Ananian CA, Ainsworth BE. 2013. Validity and reliability of the Global Physical Activity Questionnaire (GPAQ). Measurement in Physical Education and Exercise Science 17(3):221-35 DOI 10.1080/1091367X.2013.805139.

Janz KF, Broffitt B, Levy SM. 2005. Validation evidence for the Netherlands physical activity questionnaire for young children: the Iowa bone development study. Research Quarterly for Exercise and Sport 76(3):363-369 DOI 10.1080/02701367.2005.10599308.

Jefferis BJ, Sartini C, Ash S, Lennon LT, Wannamethee SG, Whincup PH. 2016. Validity of questionnaire-based assessment of sedentary behaviour and physical activity in a population-based cohort of older men; comparisons with objectively measured physical activity data. International Journal of Behavioral Nutrition and Physical Activity 13:14 DOI 10.1186/s12966-016-0338-1.

Kolbe-Alexander TL, Lambert EV, Harkins JB, Ekelund U. 2006. Comparison of two methods of measuring physical activity in South African older adults. Journal of Aging and Physical Activity 14(1):98-114.

Leatherdale ST, Laxer RE, Faulkner GE. 2014. Reliability and validity of the physical activity and sedentary behaviour measures in the COMPASS study. COMPASS Technical Report Series 2(1). Waterloo, Ontario: University of Waterloo. Available at: [www.compass.uwaterloo.ca](http://www.compass.uwaterloo.ca)

Lynch BM, Fridenrich CM, Khandwala F, Liu A, Nicholas J, Csizmadi I. 2014. Development and testing of a past year measure of

sedentary behavior: the SIT-Q. BMC Public Health 14:899.

Marshall AL, Miller YD, Burton NW, Brown WJ. 2010. Measuring total and domain-specific sitting: a study of reliability and validity. Medicine and Science in Sports and Exercise 42(6):1094-102 DOI 10.1249/MSS.0b013e3181c5ec18.

Matthews CE, Keadle SK, Sampson J, Lyden K, Bowles HR, Moore SC, Libertine A, Freedson PS, Fowke JH. 2013. Validation of a previous-day recall measure of active and sedentary behaviors. Medicine and Science in Sports and Exercise 45(8):1629-1638 DOI 10.1249/MSS.0b013e3182897690.

Mensah K, Maire A, Oppert JM, Dugas J, Charreire H, Weber C, Simon C, Nazare J-A. 2016. Assessment of sedentary behaviors and transport-related activities by questionnaire: a validation study. BMC Public Health 16:753.

Oostdam N, Van Mechelen W, Van Poppel M. 2013. Validation and responsiveness of the AQuAA for measuring physical activity in overweight and obese pregnant women. Journal of Science and Medicine in Sport 16(5):412-416 DOI 10.1016/j.jsams.2012.09.001.

Orsini N, Bellocco R, Bottai M, Hagstromer M, Sjostrom M, Pagano M, Wolk A. 2008. Validity of self-reported total physical activity questionnaire among older women. European Journal of Epidemiology 23(10):661-667 DOI 10.1007/s10654-008-9273-z.

Pettee Gabriel K, McClain JJ, Schmid KK, Storti KL, Ainsworth BE. 2011. Reliability and convergent validity of the past-week Modifiable Activity Questionnaire. Public Health Nutrition 14(3):435-42 DOI 10.1017/s1368980010002612.

Rosenberg DE, Bull FC, Marshall AL, Sallis JF, Bauman AE. 2008. Assessment of sedentary behavior with the International Physical Activity Questionnaire. Journal of Physical Activity and Health 5 Suppl 1:S30-44.

Saint-Maurice PF, Welk GJ. 2015. Validity and calibration of the Youth Activity Profile. PLoS One 10(12):e0143949. DOI:10.1371/journal.pone.0143949.

Salmon J, Owen N, Crawford D, Bauman A, Sallis JF. 2003. Physical activity and sedentary behavior: a population-based study of barriers, enjoyment, and preference. Health Psychology 22(2):178-88.

Salmon J, Campbell KJ, Crawford DA. 2006. Television viewing habits associated with obesity risk factors: a survey of Melbourne schoolchildren. Medical Journal of Australia 184(2):64-67.

Schmitz KH, Harnack L, Fulton JE, Jacobs DR, Jr., Gao S, Lytle LA et al. 2004. Reliability and validity of a brief questionnaire to assess television viewing and computer use by middle school children. Journal of School Health 74(9):370-7.

Shuval K, Kohl HW, Bernstein I, Cheng DI, Gabriel KP, Barlow CE, Yinghui L, DiPietro L. 2014. Sedentary behaviour and physical inactivity assessment in primary care: the rapid assessment disuse index (RADI) study. British Journal of Sports Medicine 48(3):250-255 DOI 10.1136/bjsports-2013-092901.

Taras HL, Sallis JF, Patterson TL, Nader PR, Nelson JA. 1989. Television's influence on children's diet and physical activity. Journal of Developmental and Behavioral Pediatrics 10(4):176-180.

Treuth MS, Sherwood NE, Butte NF, McClanahan B, Obarzanek E, Zhou A, Ayers C, Adolph A, Jordan J, Jacobs DR, Rochon J. 2003. Validity and reliability of activity measures in African-American girls for GEMS. Medicine and Science in Sports and Exercise 35(3):532-539 DOI 10.1249/01.mss.0000053702.03884.3f.

Umstattd Meyer MR, Baller SL, Mitchell SM, Trost SG. 2013. Comparison of 3 accelerometer data reduction approaches, step counts, and 2 self-report measures for estimating physical activity in free-living adults. Journal of Physical Activity and Health 10(7):1068-74.

Van Nassau F, Chau JY, Lakerveld J, Bauman AE, Van der Ploeg HP. 2015. Validity and responsiveness of four measures of occupational sitting and standing. International Journal of Behavioral Nutrition and Physical Activity 12:144 DOI 10.1186/s12966-015-0306-1.

Visser M, Koster A. 2013. Development of a questionnaire to assess sedentary time in older persons--a comparative study using accelerometry. BMC Geriatrics 13:80 DOI 10.1186/1471-2318-13-80.

Wick K, Faude O, Schwager S, Zahner L, Donath L. 2016. Deviation between self-reported and measured occupational physical activity levels in office employees: effects of age and body composition. International Archives of Occupational and Environmental Health 89(4):575-582 DOI 10.1007/s00420-015-1095-1.

Wijndaele K, I DEB, Godino JG, Lynch BM, Griffin SJ, Westgate K, Brage S. 2014. Reliability and validity of a domain-specific last 7-d sedentary time questionnaire. Medicine and Science in Sports and Exercise 46(6):1248-60 DOI 10.1249/mss.0000000000000214.

Yi SS, Bartley KF, Firestone MJ, Lee KK, Eisenhower DL. 2015. Self-reported sitting time in New York City adults, the Physical Activity and Transit Survey, 2010-2011. Preventing Chronic Disease 12:E85 DOI 10.5888/pcd12.140488.
